# Transformation of a gluteal type 1 plexiform neurofibroma into a high-grade malignant peripheral nerve sheath tumor: a rare case report

**DOI:** 10.3389/fonc.2026.1786227

**Published:** 2026-04-24

**Authors:** Hui Huang, Yang Xiao, Wei Li, Wu Wu, Yange Zhang

**Affiliations:** Department of Plastic and Burn Surgery, West China Hospital of Sichuan University, Chengdu, China

**Keywords:** case report, malignant peripheral nerve sheath tumor, malignant transformation, neurofibromatosis type 1, plexiform neurofibroma

## Abstract

Neurofibromatosis type 1 is a rare autosomal dominant disorder with heterogeneous clinical manifestations. Under certain conditions, plexiform neurofibromas may undergo malignant transformation into the more aggressive malignant peripheral nerve sheath tumor (MPNST). This case report describes a 43-year-old male with neurofibromatosis type 1 who underwent preoperative interventional vascular therapy followed by resection of a giant gluteal mass. Postoperative histopathological examination confirmed malignant transformation to MPNST. The key features of this case include a giant and rapidly progressive tumor, diagnostic challenges related to a history of trauma, tumor-related cachexia, and a multidisciplinary treatment strategy involving preoperative embolization. This case highlights the importance of regular follow-up in patients with neurofibromatosis type 1, accurate differential diagnosis, and early recognition of malignant transformation. In addition, it demonstrates the value of preoperative embolization in the management of highly vascular tumors.

## Introduction

Neurofibromatosis type 1 (NF1) is an autosomal dominant tumor predisposition disorder caused by mutations in the NF1 gene. Patients with NF1 have a significantly increased risk of malignant transformation, among which malignant peripheral nerve sheath tumor (MPNST) is the most clinically significant (1). Due to its rapid growth, marked local invasiveness, and potential for distant metastasis (2–4), MPNST has become an important cause of death in patients with NF1. Therefore, early recognition of malignant transformation in NF1 is crucial for improving patient prognosis, although it remains challenging in clinical practice (5, 6).

The early clinical manifestations of MPNST are often nonspecific, and some symptoms overlap with those of benign plexiform neurofibromas (pNF) (7). Moreover, patients with NF1 frequently present with multiple lesions, and confounding factors such as trauma or infection may obscure early warning clinical signs, further increasing the difficulty of diagnosis (8). Particularly in the rare instances where a chronic lesion that has been relatively stable for a long time rapidly progresses within a short period, recognizing this abnormal change and making an accurate assessment remain key challenges in disease management. In this study, we report a case of NF1-associated MPNST with a complex clinical course, focusing on the diagnostic challenges and treatment strategies.

## Case presentation

### Patient information

A 43-year-old male presented with a right gluteal mass of more than 40 years’ duration, with rapid enlargement over the past 2 months. The patient reported a fist-sized subcutaneous mass in the right gluteal region since childhood, accompanied by scattered cutaneous hyperpigmentation. Approximately 8 months prior to presentation, he experienced pain following a contusion to the buttock; however, there were no obvious changes in the size of the mass or the overlying skin. He was diagnosed with sciatica at a local hospital, and his symptoms improved after medication. About 2 months before admission, the gluteal mass began to enlarge rapidly without an apparent cause. This was accompanied by significant weight loss of approximately 10 kg and decreased appetite. The family history was notable, as both his mother and younger brother had neurofibromatosis type 1 (NF1).

### Clinical presentation

**Figure 1:**
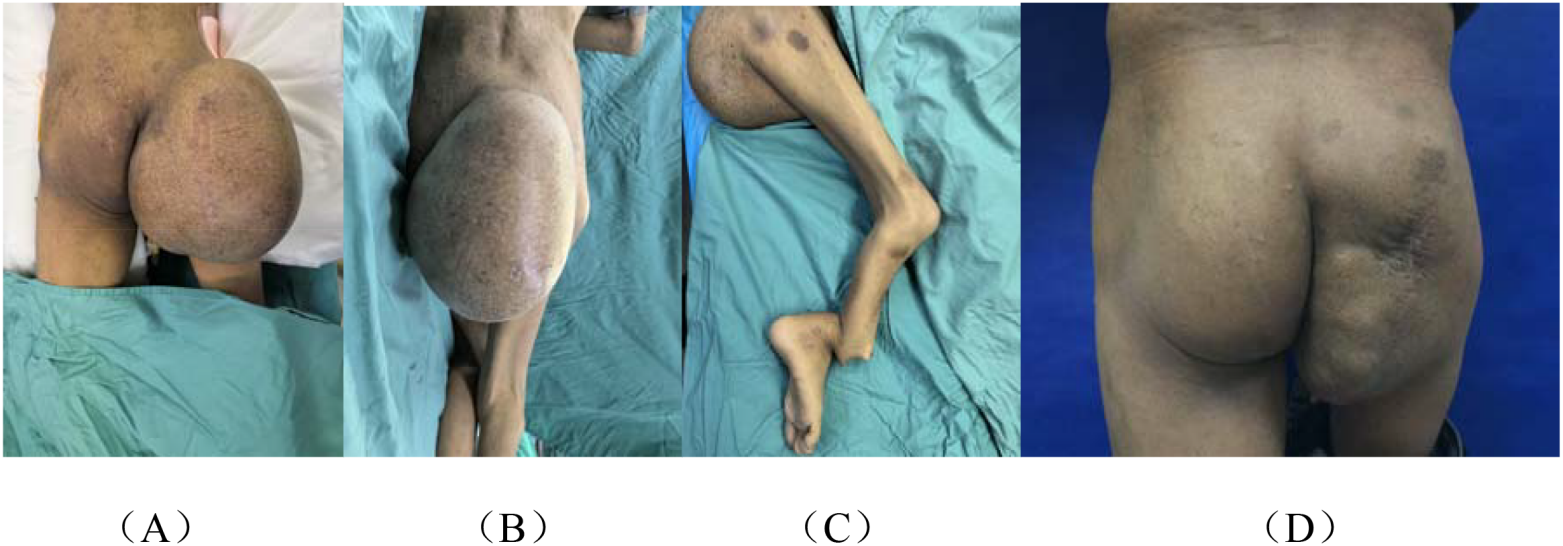
Clinical images. **(A, B)** A huge lump on the right hip. **(C)** Deformity of the right ankle joint and dislocation of the tibia. **(D)** The condition of the tumor on the patient’s buttocks after the stitches were removed.

Physical examination showed a body weight of 36 kg, with an anemic appearance and scoliosis. Multiple café-au-lait spots were scattered throughout the body. A giant mass was observed in the right gluteal region, measuring approximately 25 cm × 30 cm × 20 cm (**Figures 1A, B**). The overlying skin showed no erythema, ulceration, or purulent discharge, and there was no increase in local temperature. Musculoskeletal examination revealed deformity of the right ankle joint and severe displacement of the tibia, resulting in a limping gait and marked muscle atrophy of the right lower limb (**Figure 1C**).

### Diagnostic assessment

**Figure 2:**
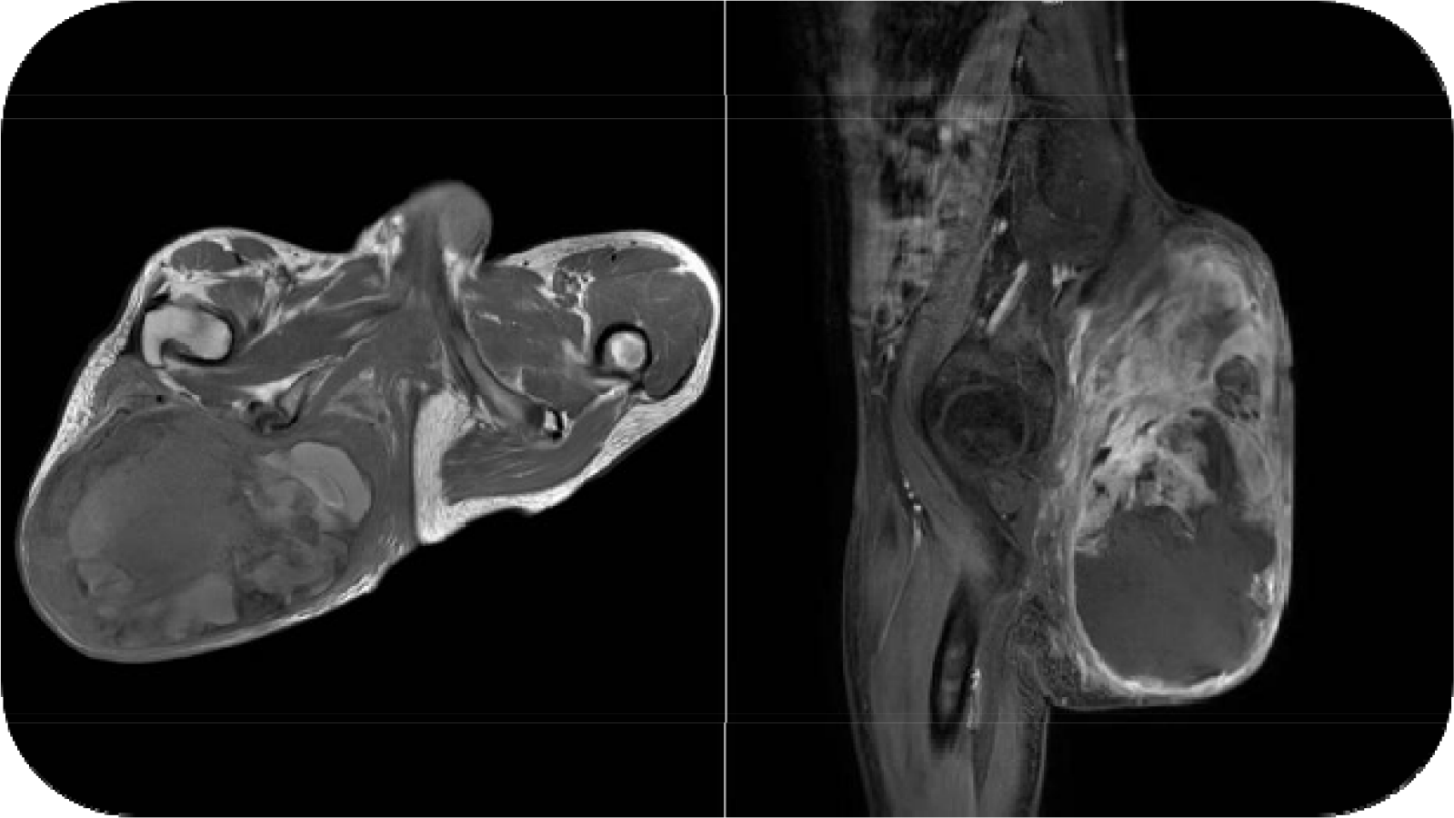
Preoperative axial and sagittal MRI views of the buttocks: A neoplastic lesion was observed in the subcutaneous tissue of the right buttock, with a maximum cross-sectional size of approximately 16.6 cm × 12.1 cm × 21.2 cm.

Subsequent imaging studies were performed. Magnetic resonance imaging (MRI) of the buttock revealed a heterogeneous mass in the right subcutaneous tissue, measuring approximately 16.6 cm × 12.1 cm × 21.2 cm at its largest dimension. The lesion showed partial enhancement on contrast-enhanced sequences, suggesting a neoplastic process (**Figure 2**). Computed tomography (CT) of the chest and abdomen demonstrated scoliosis and scattered pulmonary nodules, but no evidence of distant metastasis. Given the patient’s limited financial resources, rapid disease progression, and the absence of radiological evidence of distant metastasis, PET-CT was not performed. Based on the clinical presentation and available imaging findings, malignant transformation of a plexiform neurofibroma to MPNST was strongly suspected.

### Clinical reasoning

The diagnostic process in this case was highly challenging and required careful evaluation of both clinical findings and imaging features. A history of trauma more than 8 months earlier added complexity to the assessment. At that stage, differentiation from hemorrhage secondary to rupture of a plexiform neurofibroma needed to be considered. However, rapid tumor progression over a short period, unexplained weight loss, and a clear family history of NF1 were more consistent with malignant transformation than with simple post-traumatic hemorrhage of a benign lesion. In addition, the heterogeneous enhancement pattern observed on imaging provided further support for this diagnosis.

### Therapeutic intervention

**Figure 3:**
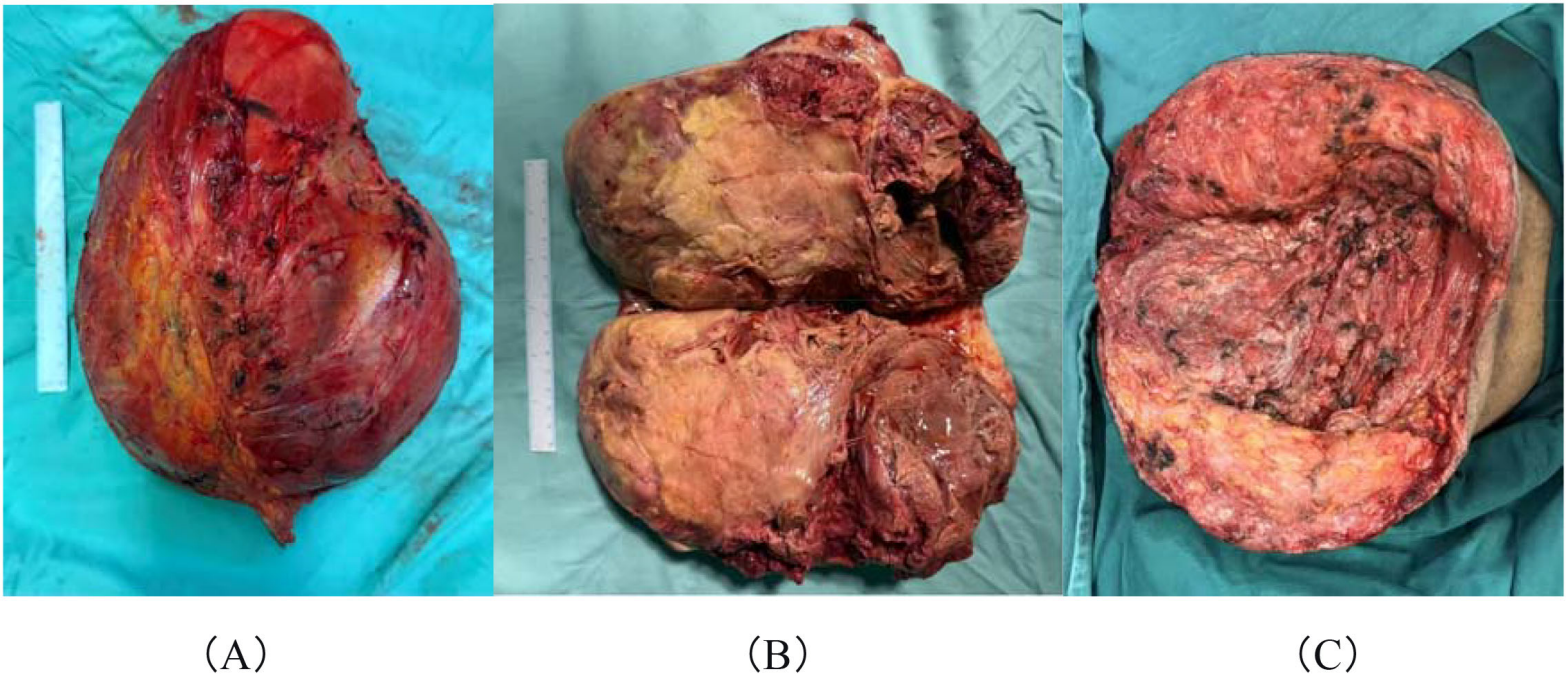
Gross examination findings. **(A)** Mass excised (view from the base of the mass). **(B)** The appearance of the mass after cutting it open. **(C)** Surgical cavity after tumor resection.

**Figure 4:**
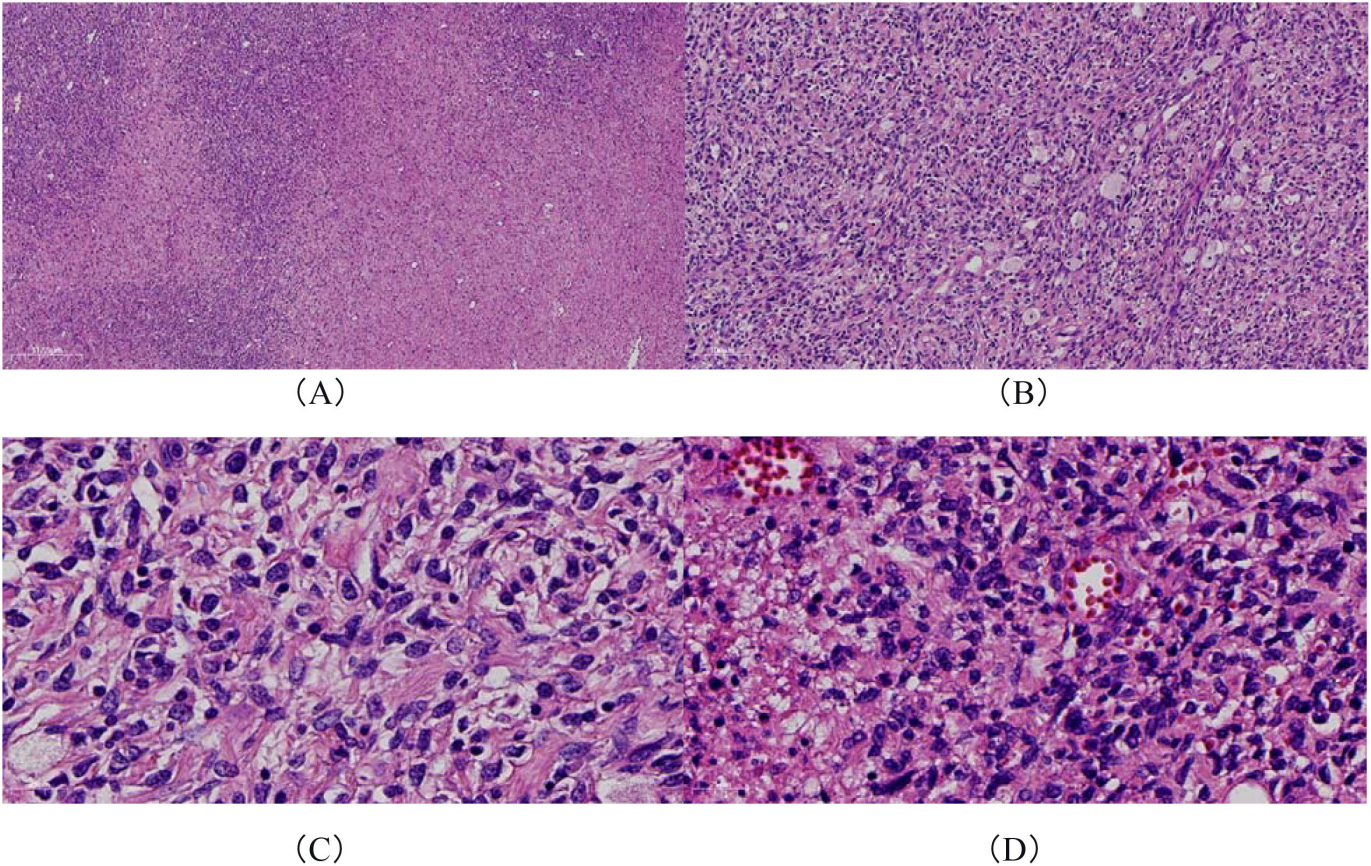
Histopathological findings. **(A)** The tumor showed high cellularity with an uneven distribution, forming a characteristic map-like pattern (H&E, ×4). **(B)** The tumor cells were densely arranged and predominantly spindle-shaped, displaying fascicular and focal whorled growth patterns (H&E, ×10). **(C, D)** Marked nuclear atypia was observed, with hyperchromatic and irregular nuclei. Mitotic figures were identified, with increased mitotic activity in certain areas. Irregular thin-walled blood vessels were present within the stroma (H&E, ×40).

Given the large tumor size and the patient’s poor general condition, preoperative vascular embolization was performed to reduce the risk of intraoperative bleeding. Under general anesthesia, the patient underwent superselective angiography of the abdominal aorta and iliac arteries, followed by coil embolization of branches of the right internal iliac artery. Resection of the right gluteal mass was then performed (**Figure 3**). Postoperative histopathological examination confirmed malignant transformation of a neurofibroma to MPNST (FNCLCC grade 3) (**Figure 4**). Immunohistochemical analysis showed focal positivity for S-100 and SOX10, retained expression of H3K27me3 and INI-1, focal positivity for CD34, and a Ki-67 proliferation index of approximately 30%. These findings support the diagnosis of MPNST.

### Follow-up and current status

**Figure 5:**
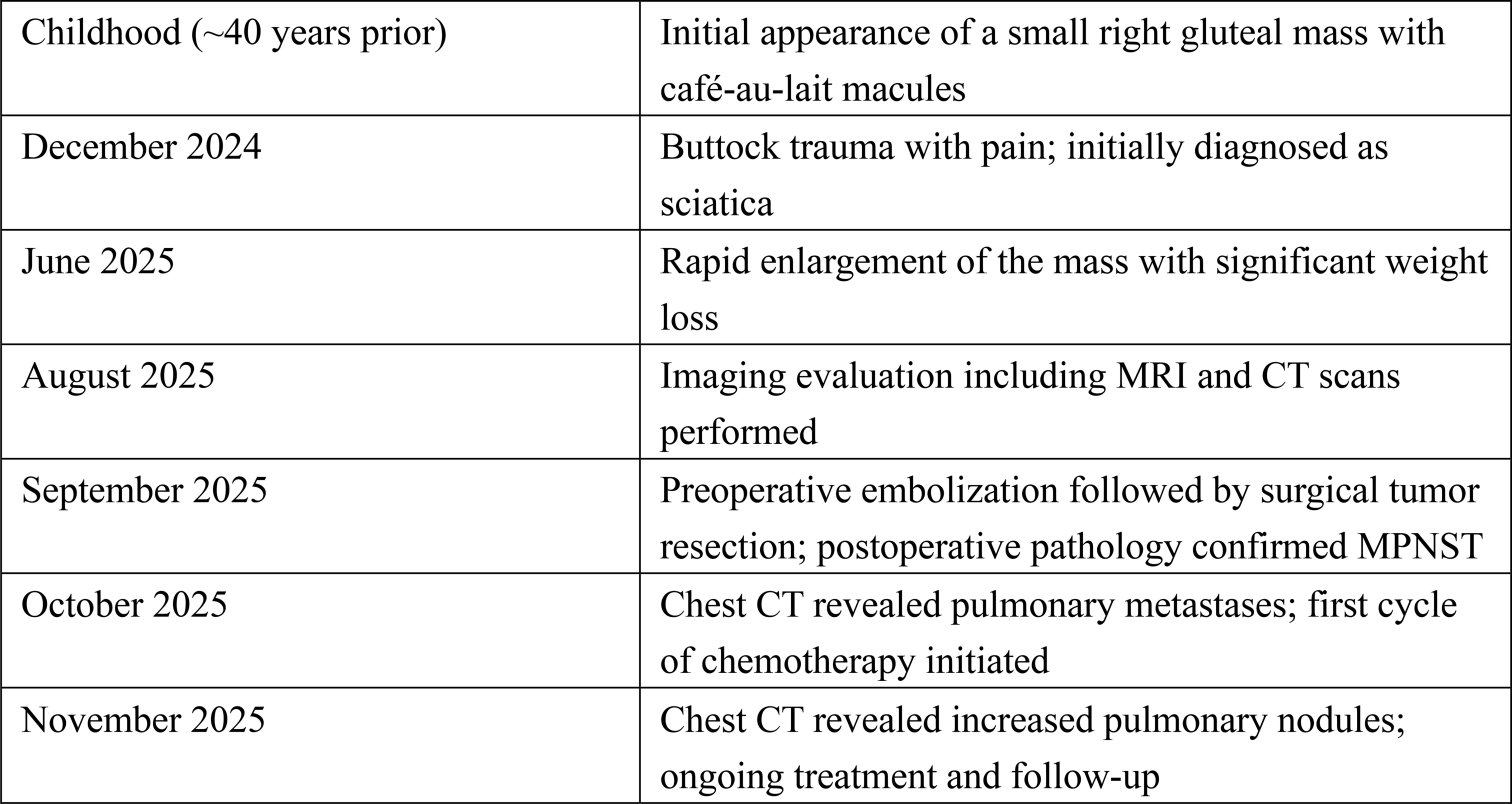
Timeline of the clinical course. Summary of key events including symptom onset, diagnostic evaluation, treatment, and short-term follow-up.

The patient was discharged on postoperative day 9, and the sutures were successfully removed on day 21 (**Figure 1D**). He reported a good subjective recovery, with a significant improvement in quality of life. Approximately 1 month after surgery, and before the initiation of chemotherapy, a follow-up chest CT revealed pulmonary metastases. The patient was subsequently referred to the oncology department and received one cycle of adjuvant chemotherapy with ifosfamide and doxorubicin (AI regimen). The treatment was well tolerated, with no significant adverse effects reported. However, repeat CT after chemotherapy showed an increased number of pulmonary nodules compared with prior imaging. As the disease progressed, the treatment strategy was further adjusted. At the last follow-up, the patient remained under active oncological treatment with close surveillance. A timeline summarizing the clinical course is presented in **Figure 5**.

### Patient perspective

The patient reported that the rapid enlargement of the mass and the associated pain before surgery caused significant anxiety and discomfort. In the early postoperative period, his symptoms were markedly relieved, and he expressed satisfaction with the surgical outcome. Despite disease progression identified during follow-up, the patient remained compliant with subsequent treatment and follow-up.

## Discussion

Malignant peripheral nerve sheath tumor (MPNST), previously referred to as neurofibrosarcoma or malignant schwannoma, is a rare, highly aggressive, and rapidly progressive soft tissue sarcoma (1, 9). Its incidence is significantly increased in patients with neurofibromatosis type 1 (NF1), with an estimated incidence of approximately 1 in 3,500 (10). Common clinical manifestations include rapid enlargement of a mass, pain, paralysis, and neurological deficits. The duration of symptoms can range from several months to several years.

Histopathologically, MPNST is usually a high-grade tumor characterized by interlacing bundles of spindle cells arranged in a herringbone or storiform pattern. Immunohistochemically, S-100 and SOX10 expression is often focal or absent, and loss of H3K27me3 expression is frequently observed. One important diagnostic feature is the presence of focal S-100 positivity (11–13). Although loss of H3K27me3 expression is highly specific for the diagnosis of MPNST, its retention does not exclude the diagnosis and can be observed in a subset of cases (14, 15). Therefore, the pathological diagnosis of MPNST requires a comprehensive evaluation that integrates histological features with immunohistochemical findings.

Additionally, NF1 is often associated with multiple soft tissue masses, and trauma may lead to hemorrhagic rupture of preexisting plexiform neurofibromas, thereby making the clinical presentation more complex. In such cases, distinguishing benign hemorrhagic rupture of a plexiform neurofibroma from early-stage MPNST is crucial, yet often challenging in clinical practice. This differentiation is based on the following features. In terms of clinical presentation, MPNST typically occurs without an obvious trigger and is characterized by persistent or progressively worsening pain, as well as rapid hardening or enlargement of the mass, often occurring over weeks to months. Of note, systemic manifestations such as unexplained weight loss, decreased appetite, and anemia, reflecting cachexia, may suggest malignant transformation. In contrast, hemorrhagic rupture is often triggered by trauma or compression and typically presents with acute swelling and pain over a short period, ranging from several hours to 1–2 days, and is frequently accompanied by neurological symptoms (16). The condition usually stabilizes thereafter. On imaging, MPNST often presents as an ill-defined mass with rich vascularity, frequently associated with bone destruction or soft tissue invasion. In contrast, ruptured neurofibromas usually demonstrate relatively well-defined margins, an intact capsule, no invasion of surrounding tissues, and no enhancement within the hemorrhagic areas (17).

In terms of imaging, MRI and CT of the lesion are essential for evaluation. Furthermore, PET-CT is commonly used to assess tumor metabolic activity, staging, and treatment response (18). However, when a tumor shows rapid progression and both the clinical presentation and imaging findings strongly suggest malignant transformation, direct surgical resection to obtain a histopathological diagnosis may also be a reasonable approach.

### Technical challenges in surgical and radiotherapeutic management of MPNST

Surgical resection remains the cornerstone of treatment for MPNST and is a key determinant of patient prognosis (19). However, due to its infiltrative growth along nerve trunks and its close proximity to adjacent neurovascular structures, achieving an R0 resection is often challenging (20). In this case, the tumor was large and highly vascularized, with a high risk of bleeding. Notably, the patient’s poor general condition and limited surgical tolerance further increased the technical difficulty of resection and raised concerns regarding the likelihood of achieving complete tumor removal. Given the high risk of bleeding and perioperative complications, superselective angiography was performed preoperatively to identify the feeding arteries, followed by coil embolization of branches of the internal iliac artery. The estimated intraoperative blood loss was 300 mL, whereas the actual blood loss was 100 mL, indicating effective bleeding control. No massive transfusion was required, which facilitated safe tumor resection. Previous studies have shown that, for large or highly vascularized soft tissue tumors, the absence of preoperative embolization is often associated with substantial intraoperative blood loss. This may lead to poor surgical field exposure and increased operative difficulty (21–23). Although direct comparative studies focusing on MPNST are limited, evidence from studies on soft tissue sarcomas indirectly supports the value of a multidisciplinary approach incorporating preoperative embolization in the management of large plexiform neurofibromas. Given the aforementioned surgical challenges, increasing attention has been directed toward alternative therapeutic strategies.

Radiotherapy, as an adjuvant treatment for MPNST, may improve local control when complete surgical resection is not achievable, thereby enhancing treatment outcomes. However, the clinical application of radiotherapy in MPNST also faces several technical challenges. In particular, for large tumors located in anatomically complex regions, adjacent critical neurovascular structures may limit accurate dose distribution, thereby increasing the risk of treatment-related toxicity. As highlighted by Ferini et al., achieving precise radiotherapy in such complex anatomical settings requires the integration of customized immobilization and advanced image-guided techniques (24). Notably, recent studies by Harikar et al. have demonstrated that with advances in radiotherapy, modern modalities such as image-guided radiotherapy (IGRT) and volumetric modulated arc therapy (VMAT) can improve dose precision, enhance tumor control, and reduce toxicity while preserving function (25). Although advanced radiotherapy techniques may offer potential benefits in selected cases, in the present case, considering the extremely large tumor size, the complex gluteal anatomy, and the patient’s poor overall condition, a treatment strategy combining preoperative embolization with surgical resection was deemed more appropriate.

In this case, the tumor was extremely large and showed rapid progression over a short period following a long phase of relative stability, a disease course that has rarely been reported. In addition, a history of trauma introduced diagnostic uncertainty, and the presence of marked systemic wasting further contributed to the complexity of the case. In terms of treatment, a multidisciplinary approach combining preoperative embolization with surgical resection was adopted. This strategy provided better conditions for the safe removal of large, highly vascularized tumors. During follow-up, pulmonary metastases were detected as early as 1 month after surgery. Moreover, the number of pulmonary nodules increased further after chemotherapy, indicating rapid disease progression. This clinical course further supports the highly aggressive nature of NF1-associated MPNST, its early tendency for distant metastasis, and its limited initial response to conventional chemotherapy (26–28). These features distinguish this case from previously reported NF1-associated MPNST cases and highlight its uniqueness in terms of clinical presentation, disease course, and management approach.

### Clinical warning signs of malignant transformation in NF1

Based on the literature and experience in this case, clinical warning signs of malignant transformation in patients with NF1 should be carefully recognized. These include rapid tumor enlargement over a short period, persistent and unrelieved pain, new-onset neurological deficits, and unexplained weight loss or cachexia. Even in the presence of an identifiable trigger, the possibility of malignant transformation should not be overlooked, particularly in patients with a relevant family history. In such situations, a comprehensive imaging evaluation should be performed promptly, and surgical biopsy may be considered when necessary. These measures may help improve diagnostic accuracy and ultimately enhance patient outcomes (7).

In addition to improving diagnostic accuracy, standardized postoperative follow-up is essential given the high risk of local recurrence and distant metastasis in MPNST, particularly pulmonary metastasis (29, 30). In clinical practice, MRI is the most important imaging modality for evaluating local recurrence (6, 31), while chest CT is used to detect pulmonary metastases. When available, PET-CT may also be considered for the assessment of systemic metastasis (32). For high-grade soft tissue sarcomas such as MPNST, follow-up is generally recommended every 3–6 months during the first 2–3 years after surgery, and every 6–12 months thereafter (33, 34). Early detection of recurrence or metastasis is critical for improving long-term outcomes.

## Conclusion

MPNST most commonly arises from the malignant transformation of plexiform neurofibromas, and its diagnosis relies on a comprehensive assessment of clinical presentation, imaging findings, and histopathology features (35). This case suggests that malignant transformation to MPNST should be strongly suspected in patients with NF1 when the following clinical features are present: rapid enlargement of a previously stable mass over a short period, persistent pain, and unexplained weight loss or anemia (7). Timely recognition of these clinical warning signs can help avoid diagnostic delay and improve patient outcomes. Additionally, this case represents a successful example of a multidisciplinary treatment approach. For large, highly vascularized soft tissue tumors, preoperative embolization can enhance surgical safety.

## Data Availability

The original contributions presented in the study are included in the article/supplementary material. Further inquiries can be directed to the corresponding author.
